# A comparative assessment between Globorisk and WHO cardiovascular disease risk scores: a population-based study

**DOI:** 10.1038/s41598-023-40820-3

**Published:** 2023-08-30

**Authors:** Azizallah Dehghan, Fatemeh Rezaei, Dagfinn Aune

**Affiliations:** 1 Noncommunicable Diseases Research Center, Fasa University of Medical Sciences, Fasa, Iran; 2 Research Center for Social Determinants of Health, Jahrom University of Medical Sciences, Jahrom, Iran; 3 Department of Epidemiology and Biostatistics, School of Public Health, Imperial College London, London, UK; 4 Department of Nutrition, Oslo New University College, Oslo, Norway

**Keywords:** Diseases, Health care, Medical research, Risk factors

## Abstract

The Globorisk and WHO cardiovascular risk prediction models are country-specific and region-specific, respectively. The goal of this study was to assess the agreement and correlation between the WHO and Globorisk 10-year cardiovascular disease risk prediction models. The baseline data of 6796 individuals aged 40–74 years who participated in the Fasa cohort study without a history of cardiovascular disease or stroke at baseline were included. In the WHO and Globorisk models scores were calculated using age, sex, systolic blood pressure (SBP), current smoking, diabetes, and total cholesterol for laboratory-based risk and age, sex, SBP, current smoking, and body mass index (BMI) for non-laboratory-based risk (office-based or BMI-based). In Globorisk and WHO risk agreement across risk categories (low, moderate, and high) was examined using the kappa statistic. Also, Pearson correlation coefficients and scatter plots were used to assess the correlation between Globorisk and WHO models. Bland–Altman plots were presented for determination agreement between Globorisk and WHO risk scores in individual’s level. In laboratory-based models, agreement across categories was substantial in the overall population (kappa values: 0.75) and also for females (kappa values: 0.74) and males (kappa values: 0.76), when evaluated separately. In non-laboratory-based models, agreement across categories was substantial for the whole population (kappa values: 0.78), and almost perfect for among males (kappa values: 0.82) and substantial for females (kappa values: 0.73). The results showed a very strong positive correlation (r ≥ 0.95) between WHO and Globorisk laboratory-based scores for the whole population, males, and females and also a very strong positive correlation (r > 0.95) between WHO and Globorisk non-laboratory-based scores for the whole population, males, and females. In the laboratory-based models, the limit of agreements was better in males (95%CI 2.1 to − 4.2%) than females (95%CI 4.3 to − 7.3%). Also, in the non-laboratory-based models, the limit of agreements was better in males (95%CI 2.9 to − 4.0%) than females (95%CI 3.2 to − 6.1%). There was a good agreement between both the laboratory-based and the non-laboratory-based WHO models and the Globorisk models. The correlation between two models was very strongly positive. However, in the Globorisk models, more people were in high-risk group than in the WHO models. The scatter plots and Bland–Altman plots showed systematic differences between the two scores that vary according to the level of risk. So, for these models may be necessary to modify the cut points of risk groups. The validity of these models must be determined for this population.

## Introduction

One of the world’s top three causes of mortality are cardiovascular diseases (CVDs). In 2019, of the 55 million deaths worldwide, non-communicable diseases (NCDs) accounted for 71%. Cardiovascular diseases (CVDs) contributed the largest number of deaths worldwide (17.9 million) among NCDs^1^. According to the Global Burden of CVD, years lived with disability increased from 17.7 million to 34.4 million between 1990 and 2019^2^. The burden of CVDs is particularly high in low- and middle-income countries (LMICs), and because of limited human and economic resources deaths occurs at younger ages than in high-income countries (HIC)^3, 4^. HICs have reported a decrease in CVD mortality rates over the last decades but 80% of the morbidity burden and 50% of the mortality burden due to CVD is reported to occur in LMICs including the Eastern Mediterranean Region (EMR)^5^.

Recently, many tools and clinical practice guidelines have been developed for CVD risk prediction, including the Framingham risk score, European Society of Cardiology/European Society of Hypertension guidelines, American College of Cardiology and American Heart Association (ACC/AHA guidelines, Systematic COronary Risk Evaluation (SCORE), and SCORE2^6–10^. These tools have shown benefit for risk prediction, however, they were developed and have mainly been tested in Europe and America^11^. Because the levels of CVD predictors vary across populations, CVD risk prediction models that have been developed for specific countries cannot automatically be used in other countries^12, 13^. In the recent decade, two CVD risk equations; WHO and Globorisk were developed that are more specific to each population.

In 2015, Hajifathalian et al. developed the Globorisk CVD risk prediction equations. Globorisk is a new CVD risk calculator that is country-specific. Globorisk has been validated and calibrated using data from 182 countries and its external validity has been examined in different regions^14, 15^. In 2019, WHO revised previous CVD risk prediction models that were created in 2007. The new WHO CVD risk equations estimated risk for 21 regions of GBD. Also, revised WHO models estimate risk for LMICs^16^.

WHO and Globorisk CVD risk prediction equations have a laboratory-based and non-laboratory-based version. Laboratory-based models estimate CVD risk using age, sex, SBP, smoking, diabetes, and cholesterol. Non-laboratory-based estimated CVD risk using age, sex, smoking, SBP, and BMI^15, 16^.

It is important to evaluate the agreement between two 10-year risk prediction models of CVDs, including the WHO model (a region-specific model) and Globorisk (a country-specific model). Some previous studies in Iran determined the 10-year risk of CVDs with Framingham and WHO models^17–19^. However, no study has been conducted to evaluate the agreement and correlation between Globorisk and WHO risk prediction models. Therefore, the present study was conducted with the aim of examining the agreement between the laboratory-based versions of the WHO and Globorisk models, as well as the agreement between the non-laboratory-based versions of these two models in a large Iranian population.

## Methods

This cross-sectional study is a part of Fasa cohort study. The details of the Fasa cohort study have been published previously^20, 21^. Briefly, the study population in the Fasa cohort study was 10,138 persons aged ≥ 35 years old, resident in the Sheshdeh region in Fasa county. Data were collected from 2015 to 2016. Before beginning the study, interviewers were trained to collect data. Trained interviewers collected and registered demographic characteristics, medical history (diabetes, cardiovascular disease, cancer, and etc.), physical activity, smoking status, opium use and alcohol drinking.

To record the anthropometric characteristics, the trained staff measured the height, weight and waist circumference of the participants. Laboratory staff took samples for biochemical tests, including blood sugar and blood lipids including cholesterol, triglycerides (TG), high density lipoprotein (HDL), and low density lipoprotein (LDL). In this study, people who had a history of cardiovascular diseases and stroke were excluded. Also, because the 10-year CVD risk of Globorisk equations was created for people aged ≥ 40 years, participants < 40 years old were excluded. WHO CVD risk equations were defined for persons aged 40–74 years, participants > 74 years old were additionally excluded. Finally, 6796 persons aged 40–74 who had no history of CVD and stroke were included in this study.

### CVD risk assessment

In this study, fatal plus non-fatal WHO and Globorisk CVDs risk prediction models were used to determine 10-year CVD risk. For WHO and Globorisk models, cardiovascular outcomes are a 10-year risk of fatal and nonfatal CVD, CHD, or stroke^22^. WHO and Globorisk equations have two versions; laboratory-based and non-laboratory-based. In the WHO laboratory-based model, CVD risk determined by factors including age, sex, smoking status, diabetes status, SBP and cholesterol (mmol/L). In the WHO non-laboratory-based model, cholesterol and diabetes is replaced by BMI and risk is calculated based on age, sex, smoking status, SBP, and BMI. CVD risk in WHO models is determined according 21 regions of GBD. According to GBD, Iran is in North Africa and Middle East region. Afghanistan, Algeria, Bahrain, Egypt, Iraq, Jordan, Kuwait, Lebanon, Libya, Morocco, Occupied Palestinian territory, Oman, Qatar, Saudi Arabia, Sudan, Syrian Arab Republic, Tunisia, Turkey, United Arab Emirates, and Yemen are included in this region^22^.

Globorisk is country-specific model. Globorisk also has laboratory-based and non-laboratory-based versions. In the laboratory-based version, risk factors include age, sex, SBP, smoking status, diabetes status, and cholesterol (mmol/L). The office-based version includes age, sex, SBP, smoking status and BMI^15^.

In the Fasa cohort study, all measurements were done according PERSIAN cohort guidelines^23^. Smoking status was determined by questions about smoking. For biochemical index measures, each person had to have fasted for 12–16 h. Diabetes was determined by self-reported history of diabetes, treatment or fasting blood sugar ≥ 126 mg/dl. Blood pressure was measured two times with an interval of 5 min after a five-minute rest in sitting position using a standard calibrated sphygmomanometer and the mean of the two measurements was recorded. Weight (kg) and height (m) were measured and BMI calculated (weight in kilograms divided by height in meters squared).

### Ethics approval and consent to participate

This study was approved by the Ethics Committee of Jahrom University of Medical Sciences (IR.JUMS.REC.1400.071). The informed consent of participants was obtained if they intended to participate in the study and all information of the participants was collected anonymously. Also, all methods were carried out in accordance with relevant guidelines and regulations.

### Statistical analysis

Percentages were used for categorical variables and means ± standard deviations (SDs) were used for continuous variables were used. Chi-square and t-test were used to test for differences across categories. The analysis was done using different methods according to whether the risk is grouped or quantitative. Globorisk and WHO risk scores were divided into three categorized (low, moderate, and high) and percent agreement and kappa statistics were estimated. Kappa statics range is − 1 to 1. However, most is between 0 to 1. According to the suggestion of Landis and Koch, the strength of agreement was interpreted as follows: kappa statistic ≤ 0 be considered 'poor' agreement, kappa statistic = 0.00–0.20 be considered 'slight' agreement, kappa statistic = 0.21–0.40 be considered 'fair' agreement, kappa statistic = 0.41–0.60 be considered 'moderate' agreement, kappa statistic = 0.61–0.80 be considered ' substantial', and kappa statistic = 0.81–1.00 be considered 'almost perfect'^24^.

In WHO risk models, risk categorized as < 5%, 5% to < 10%, 10% to < 20%, 20% to < 30% and ≥ 30% was defined as very low-, low-, moderate-, high-, and very high-risk^16^. For comparison WHO and Globorisk models, WHO risk scores was categorized in three groups < 10%, 10–< 20% and ≥ 20% which were considered low-, moderate-, and high-risk. In Globorisk models, risk grouping has not been specified the same way as other CVDs risk prediction models. In the study by Ueda et al. from an Iranian population a threshold ≥ 20% risk was considered a high-risk group^15^. So, In Globorisk models, risk categorized as < 10%, 10% to < 20%, and ≥ 20% were considered low-, moderate-, and high-risk.

The correlation between WHO and Globorisk models was calculated using correlation coefficients and scatter plots. A scatter plot shows the relationship between two quantitative variables measured for the same people. The general pattern of a scatter plot can be described by the direction, form and strength of the association. The correlation coefficient measures the strength of that association. The value of the correlation coefficient ranges from − 1 to 1^25^. According to the suggestion of Evans, a correlation coefficient between two variables is considered to have a very weak correlation (0.00–< 0.20), weak correlation (0.20–< 0.40), moderate correlation (0.40–< 0.60), strong correlation (0.60–< 0.80), and very strong correlation (0.80–1.0)^26^.

Bland–Altman plots were presented for determination agreement between risk scores at the individual level. This method was used to assess agreement between two quantitative measurements. A Bland–Altman plot is a scatter plot that plots the difference between two paired of measurements on the Y axis and the mean of the two measurements on the X axis^27^.

All statistical analyses were conducted using Statistical Package for Social Science (IBM SPSS Statistics for Windows, Version 23.0) and Stata Statistical Software (Stata for windows, Version 14). P-values < 0.05 were considered as significant.

## Results

In this study, 6796 participants were included. Table 1 shows a summary of participants’ characteristics. In brief, 53.5% were female, and mean (SD) age of the participants was 51.0 (7.8) years. Also, 52.3% were illiterate. The prevalence of smoking was higher in males than in females, while the prevalence of hypertension and diabetes was higher in females than in males. SBP, DPB, HDL, cholesterol, and BMI was higher in females than males.

**Table 1:** Reporting of the participants’ characteristics. DBP, diastolic blood pressure; SBP: systolic blood pressure; HDL: high density lipoprotein; Chol: cholesterol; BMI: body mass index, * chi-square test, ** t-test.

Variables	Total (n = 6796)	Males (n = 3158)	Females (n = 3638)	P-value
	N (%)	N (%)	N (%)	
Age range (years), (Mean ± SD)	51.0 ± 7.8	51.1 ± 7.9	51.0 ± 7.7	0.76**
Education level				
Illiterate	3557 (52.3)	1234(39.1)	2323 (63.9)	< 0.001*
≤ diploma	3140 (46.2)	1840 (58.3)	1300 (35.7)	
University	99 (1.5)	84 (2.7)	15 (0.4)	
Smoking (current)				
No	5446 (80.1)	1894 (60.0)	3552 (97.6)	< 0.001*
Yes	1350 (19.9)	1264 (40.0)	86 (2.4)	
Hypertension				
No	5508 (81.0)	2733 (89.7)	2675 (73.5)	< 0.001*
Yes	1288 (19.0)	325 (10.3)	963 (26.5)	
Diabetes				
No	5924 (87.2)	2907 (92.1)	3017 (82.9)	< 0.001*
Yes	872 (12.8)	251 (7.9)	621 (17.1)	
DBP (Mean mmHg ± SD)	75.0 ± 11.8	74.6 ± 11.7	75.4 ± 11.9	0.003**
SBP (Mean mmHg ± SD)	112.4 ± 18.4	111.2 ± 17.5	113.4 ± 19.1	< 0.001**
HDL (Mean mmol/l ± SD)	1.3 ± 0.4	1.2 ± 0.4	1.4 ± 0.4	< 0.001**
Chol (Mean mmol/l ± SD)	4.9 ± 1.0	4.7 ± 0.9	5.0 ± 1.0	< 0.001**
BMI (Mean kg/m^2^ ± SD)	25.6 ± 4.8	24.2 ± 4.5	26.8 ± 4.8	< 0.001**

The mean of laboratory-based WHO risk score was 7.4 ± 5.4 and the mean of laboratory-based Globorisk score was 6.1 ± 6.9. Also, the mean of non-laboratory-based WHO and Globorisk risk scores was 7.2 ± 4.9 and 6.2 ± 6.4, respectively. In the WHO and Globorisk CVDs risk scores, the mean laboratory-based and non-laboratory-based models was higher in males than females (Table 2).

**Table 2:** The distribution of the 10-year WHO and Globorisk CVD risk scores.

CVD risk prediction models	Total (n = 6796)	Males (n = 3158)	Females (n = 3638)	**P-value**
	Mean ± SD	Mean ± SD	Mean ± SD	
Laboratory-based WHO CVDs risk score	7.4 ± 5.4	7.9 ± 5.8	6.9 ± 5.1	< 0.001**
Laboratory-based Globorisk CVDs risk score	6.1 ± 6.9	6.8 ± 6.2	5.4 ± 7.4	< 0.001**
Non-laboratory-based WHO CVDs risk score	7.2 ± 4.9	7.8 ± 5.4	6.6 ± 4.4	< 0.001**
Non-laboratory-based Globorisk CVDs risk score	6.2 ± 6.4	7.3 ± 6.3	5.2 ± 6.3	< 0.001**

**Figure 1:**
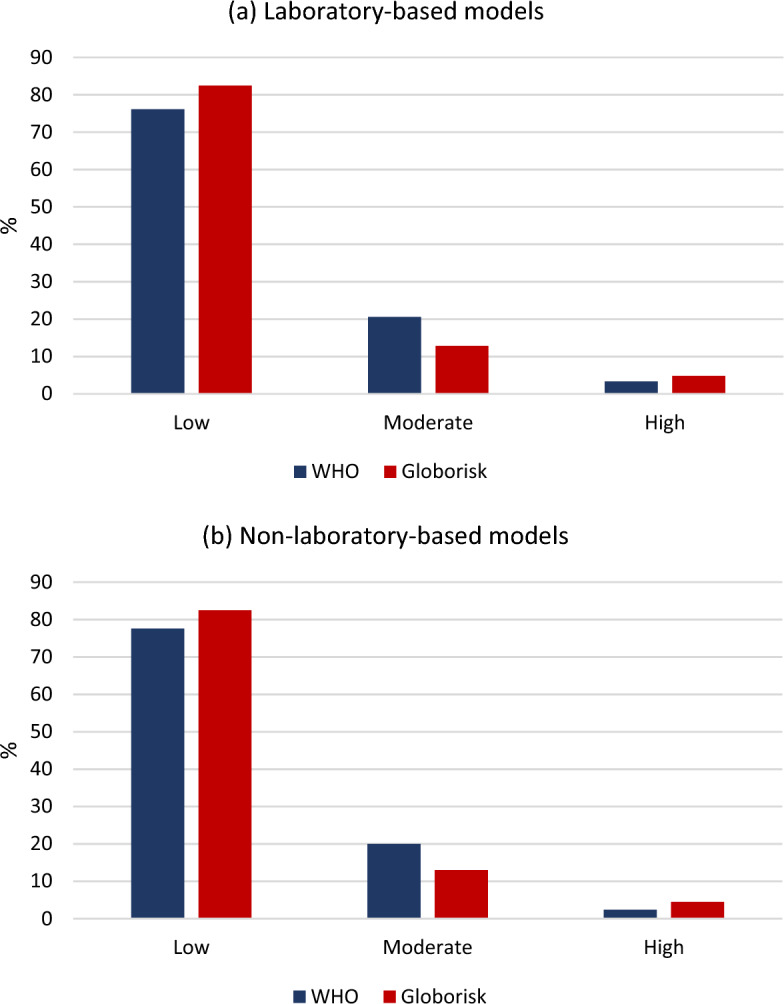
The percentage of the 10-years WHO and Globorisk CVD risks classified (**a**) laboratory-based models; (**b**) non-laboratory-based models.

Figure 1 showed that in the WHO and Globorisk laboratory-based models 3.3% and 4.8% were in high-risk group, respectively. Also, in the WHO and Globorisk non-laboratory-based models 2.4% and 4.7% were in high-risk group, respectively.

Tables 3 and 4 shows the agreement of grouped risk between the WHO and Globorisk risk scores according to the laboratory-based and non-laboratory-based models, respectively. In the total population, agreement between the laboratory-based WHO and Globorisk risk groups was 91.4% (kappa = 0.75). The agreement was almost the same in females (kappa = 0.74) and males (kappa = 0.76).

**Table 3:** Agreement between the laboratory-based WHO and Globorisk cardiovascular disease risk scores models according to the grouped risk.

Laboratory-based Globorisk risk category	Laboratory-based WHO risk category				Agreement (%)	Kappa* (SE**)
	Low	Moderate	High	Total		
All population						
Low	5168	429	0	5597	91.4	0.75 (0.009)
Moderate	1	845	26	872		
High	0	126	201	327		
Total	5169	1400	227	6796		
All males						
Low	2316	214	0	2530	91.1	0.76(0.013)
Moderate	1	453	25	479		
High	0	40	109	149		
Total	2317	707	134	3158		
All females						
Low	2952	215	0	3067	94.4	0.74 (0.013)
Moderate	0	392	1	393		
High	0	86	92	178		
Total	2952	693	93	3638		

*≤ 0 “poor”, 0.00–0.20 “slight”, 0.21–0.40 “fair”, 0.41–0.60 “moderate”, 0.61–0.80 “substantial”, 0.81–1.00 “almost perfect”.

**Standard Error.

**Table 4:** Agreement between the non-laboratory-based WHO and Globorisk cardiovascular disease risk scores models according to the grouped risk.

Non-laboratory-based Globorisk risk category	Non-laboratory-based WHO risk category				Agreement (%)	Kappa* (SE**)
	Low	Moderate	High	Total		
All population						
Low	5274	335	0	5609	92.7	0.78 (0.009)
Moderate	0	871	10	881		
High	0	154	152	306		
Total	5274	1360	162	6796		
All males						
Low	2321	163	0	2484	92.9	0.82(0.011)
Moderate	0	509	10	519		
High	0	50	105	155		
Total	2321	722	115	3158		
All females						
Low	2953	172	0	3125	92.4	0.73 (0.014)
Moderate	0	362	0	362		
High	0	104	47	151		
Total	2953	638	47	3638		

* ≤ 0 “poor”, 0.00–0.20 “slight”, 0.21–0.40 “fair”, 0.41–0.60 “moderate”, 0.61–0.80 “substantial”, 0.81–1.00 “almost perfect”.

**Standard Error.

In the total population, agreement between the non-laboratory-based WHO and Globorisk risk groups was 92.6% (kappa = 0.78). The agreement was better in males than females (kappa: 0.82 vs. 0.73).

**Figure 2:**
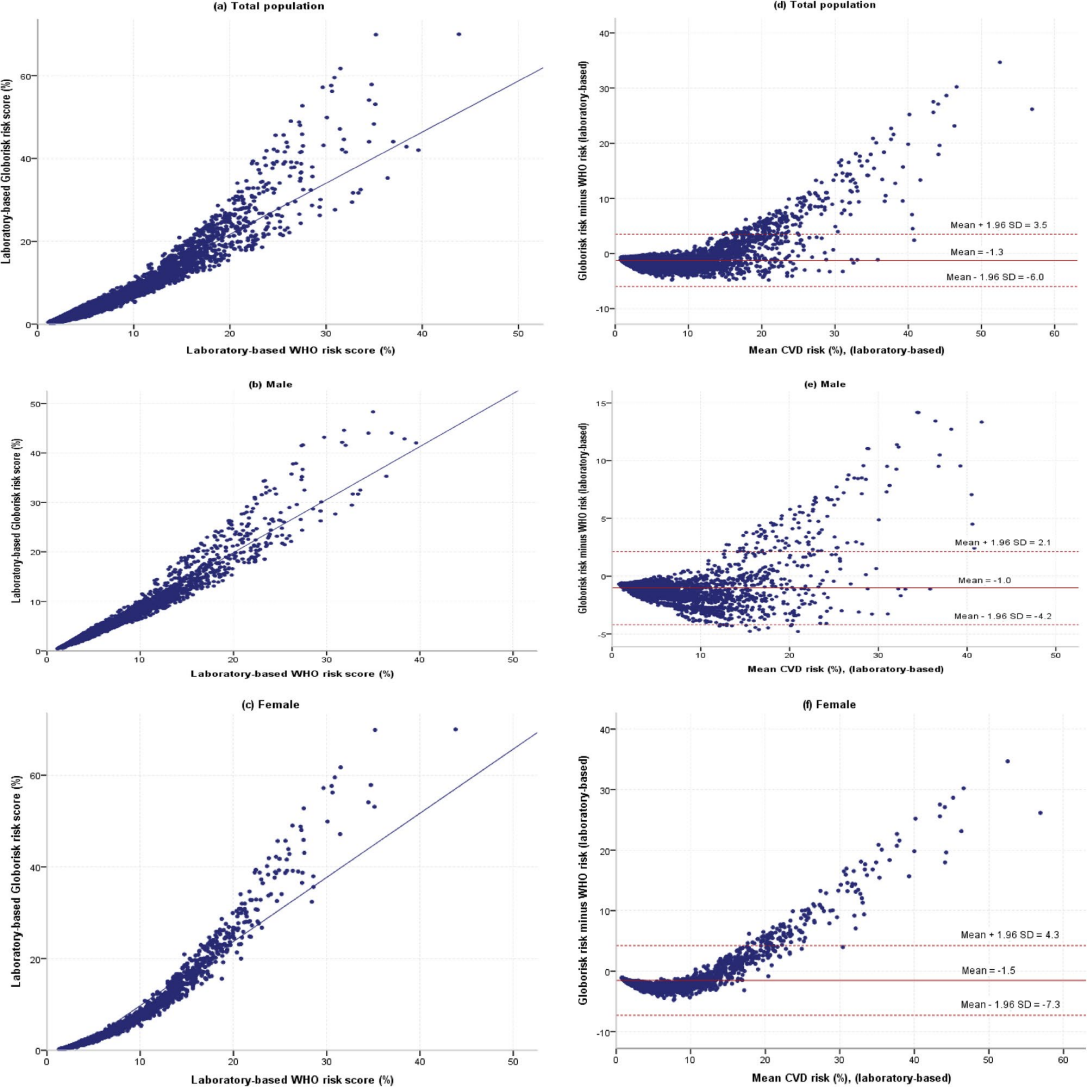
Scatter plots and Bland–Altman plots of individual-level risk of laboratory-based WHO and Globorisk cardiovascular disease risk scores models. (**a**) scatter plot for total population, (**b**) scatter plot for males, (**c**) scatter plot for females, (**d**) Bland–Altman plot for total population, (**e**) Bland–Altman plot for males, (**f**) Bland–Altman plot for females.

**Figure 3:**
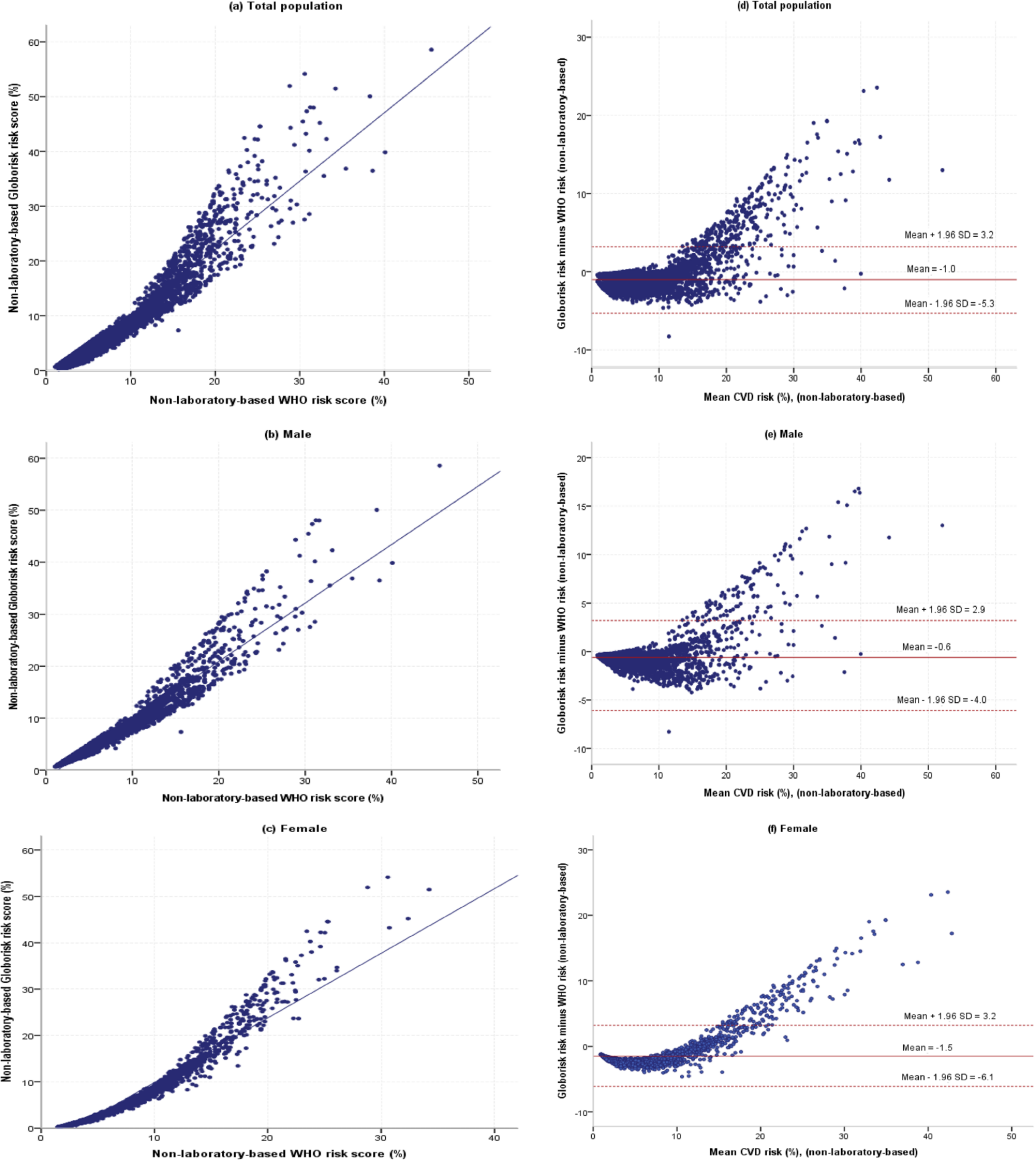
Scatter plots and Bland–Altman plots of individual-level risk of non-laboratory-based WHO and Globorisk cardiovascular disease risk scores models. (**a**) scatter plot for total population, (**b**) scatter plot for males, (**c**) scatter plot for females, (**d**) Bland–Altman plot for total population, (**e**) Bland–Altman plot for males, (**f**) Bland–Altman plot for females.

Correlation coefficients between the WHO and Globorisk risk scores are shown in Table 5. In the overall population and in males and females separately, the correlations between laboratory-based WHO and Globorisk CVD risk scores were very strong. There were significant positive correlations between the laboratory-based WHO and Globorisk risk scores in males and females. Also, in the overall population and in males and females separately, the correlations between non-laboratory-based WHO and Globorisk CVD risk scores were very strong. There were significant positive correlations between the non-laboratory-based WHO and Globorisk risk scores in males and females (Figs. 2 and 3).

**Table 5:** Correlation coefficients between WHO and Globorisk cardiovascular disease risk scores models according to laboratory-based and non-laboratory-based model.

	N	r* (95% CI)	p-value	Comment**
Laboratory-based				
Male	3158	0.98 (0.98–0.98)	< 0.001	Very strong
Female	3638	0.96 (0.96–0.96)	< 0.001	Very strong
Total	6796	0.95 (0.95–0.95)	< 0.001	Very strong
Non-laboratory-based				
Male	3158	0.97 (0.97–0.97)	< 0.001	Very strong
Female	3638	0.97 (0.97–0.97)	< 0.001	Very strong
Total	6796	0.96 (0.96–0.96)	< 0.001	Very strong

*Correlation coefficient.

**0.00–< 0.20 “very weak”, 0.20–< 0.40 “weak”, 0.40–< 0.60 “moderate”, 0.60–< 0.80 “strong”, 0.80–1.0 “very strong”.

Bland–Altman plots which show the agreement between the Globorisk and WHO risk scores on the individual level according to laboratory-based and non-laboratory-based models are shown in Figs. 2 and 3. In laboratory-based models, the limit of agreement was better for males than females (2.1 to − 4.2% vs. 4.3 to − 7.3%). Also, in non-laboratory-based models, the limit of agreement was better for males than females (2.9 to − 4.0% vs. 3.2 to − 6.1%) (Table 5).

## Discussion

In this study, two CVD risk prediction models were assessed including WHO risk model which is region-specific and Globorisk that is country-specific. To our knowledge, this study is the first study to evaluate and compare laboratory-based WHO and Globorisk and also non-laboratory-based versions of WHO and Globorisk separately in a large population. The agreement and correlation beetween these modeles were determined according to laboratory-based and non-laboratory-based models, separately.

Recently for this population, the agreement between laboratory-based and office-based Globorisk and also the agreement between laboratory-based and non-laboratory-based WHO CVD risk were estimated separately^28, 29^. The results showed that there was substantial agreement between WHO and Globorisk laboratory-based risk grouping and also between WHO and Globorisk non-laboratory-based risk grouping. Very strong positive correlations were observed between the WHO and Globorisk laboratory-based risk scores and also between the WHO and Globorisk non-laboratory-based risk scores.

The results showed substantial agreement between Globorisk and WHO risk grouping. Other studies determined agreement between CVDs risk models. Rezaei et al. assessed the agreement between laboratory-based and non-laboratory-based versions of the Framingham risk score and WHO risk equations in the Pars cohort and found lower agreement between the two than in the current analysis^18, 19^. In Bangladesh the agreement between non-laboratory-based Framingham risk score, Globorisk and WHO/ISH models was estimated, and was also lower than in the current study^30^. However, we are not aware of any studies that have assessed the agreement between the laboratory-based versions of the WHO and Globorisk models and the non-laboratory-based versions of the two models separately.

The results from this study showed that 3.3% when using the WHO laboratory-based model and 2.4% when using the WHO non-laboratory-based model were in the high risk group (≥ 20%). In another study, 5.0% and 4.7% of participants were in the the high-risk group based on the WHO laboratory-based model and the WHO non-laboratory-based model, respectively^19^. Although both studies were conducted in Iran using the revised WHO version, due to the difference in the population structure and risk factors, the risk classification differed slightly in the two studies.

Another CVD risk assessment tool that was used was the Globorisk equations. The results showed that 4.8% and 4.5% in the Globorisk laboratory- based model and non-laboratory-based model were in the high- risk group (≥ 20%), respectively. In Malaysia, fewer people were in the low-risk group, likely reflecting differences in the distribution of risk factors in that population compared to the current Iranian study. In the Globorisk laboratory-based model, 45% had a risk of < 10%, 14% ≥ 20%, and 11% ≥ 30%, and in the non-laboratory-based model 51.1% had a risk of < 10%, 11% ≥ 20%, and 4.9% ≥ 30%^31^.

In this study, the agreement between the laboratory-based WHO and Globorisk groups risk was substantial both overall and in females and males separately. The non-laboratory-based models is a highly effective screening tool at lower cost in settings with limited resources where laboratory tests are unreachable or expensive^32^. In Bangladesh, the concordance between non-laboratory-based World Health Organization/International Society of Hypertension (WHO/ISH) and Globorisk CVD risk prediction models was 0.37^30^, which was very different when compared to our study, where agreement was substantial. One explanation could be that the study from Bangladesh used the 2007 version of WHO/ISH risk model, while we used the revised WHO risk model from 2019^16^. In addition, differences in risk factor levels between the populations may also have contributed to differences in agreement.

In this study, there was a very strong positive correlation between Globorisk and WHO risk scores. On the other hand, in both laboratoty-based and non-laboratory-based models, there were more high-risk individuals in the Globorisk model than WHO model. Thus, for the important function of identifying people at elevated risk for interventions the two scores perform differently. So, Bland–Altman plots were used for determination agreement between risk scores. The results showed that in laboratory-based and also in non-laboratory-based models the agreement between the two risk scores was better among males than females. In a study carried out in Iran, the agreement between laboratory-based and BMI-based Framingham models was examined with Bland–Altman plot and showed that the agreement between the two risk scores was better among the yonger females and males and was wider among the older males^18^. A study in Peru showed that there were no substantial differences between the mean CVD risk computed with the laboratory-based model and the non-laboratory-based model^33^. It is important to mention that acceptable limits should be defined based on various factors, including clinical and biological factors, as well as other considerations that have already been defined^27, 34^, but we had no information about these factors.

Totally, this study showed that for men the limit of agreement was appropriate especially in low-risk scores. For females, although the limit of agreement was wider statistically in the laboratory and non-laboratory-based models, it seems that these differences are not very important clinically. The scatter plots and Bland–Altman plots demonstrate systematic differences between the two scores that vary according to the level of risk and are more problematic in females than in males. Almost all females at the lower end of moderate risk by WHO are classified as low risk by Globorisk. The majority of females at the lower end of high risk on Globorisk models are only moderate risk according to WHO models. This is completely consistent with the limited overlap of these categories reported in Tables 3 and 4. So, it may be necessary to modify the cut points of risk groups for the two models. However, for this population, modification of the risk grouping cut-point was not the objective of this study.

The Fasa PERSIAN cohort study has various ethnicities including Fars, Arab, and Turk that have different behaviors and socioeconomic status. Differences in ethnicity, socioeconomic status, and genetics of people in various geographic regions may also contribute to differences in CVD risk estimates. It is important to mention that most of the studies determined the agreement of the Framingham model with other models, and until the writing of this manuscript, the authors have not seen other papers that evaluated the agreement and correlation of the Globorisk model with the WHO updated version from 2019.

### Strengths and limitations

The main strength of this study was the large sample and the use of carefully collected data from a population-based study. The major limitation of the current study is the cross-sectional design using baseline data of a cohort study, to validate both laboratory-based and non-laboratory-based versions of the Globorisk and WHO risk models. Further studies using a longitudinal design and with longer follow-up is needed. Another limitation is that we only determined the agreement between the models. Further analyses using CVD incidence will allow us to evaluate how well these scores actually predict CVD outcomes.

## Conclusion

In this study, an acceptable agreement was observed between the classified risk of laboratory-based WHO and Globorisk models and the non-laboratory-based WHO and Globorisk models. Also, strong positive correlations were observed between the risk scores of these models. However, it should be noted that although a good agreement was observed between the models, a large number of people who were classified as moderate risk by the WHO models were low-risk and high-risk according to Globorisk models. Therefore, it may be necessary to modify the cut points of risk groups for the two models. For WHO and Globorisk models, the ability to determine risk groups must be confirmed separately. Further longitudinal studies with 10-year follow-up are needed to assess actual fatal plus non-fatal CVD events.

## Data Availability

Data are available to researchers upon the reasonable request by directly contacting the corresponding author.

## Abbreviations

CVD: Cardiovascular Disease
WHO: World Health Organization
SBP: Systolic Blood Pressure
DBP: Diastolic Blood Pressure
HDL: High-Density Lipoprotein
Chol: Cholesterol
BMI: Body Mass Index
NCD: Non-communicable disease
LMIC: Low- and middle-income country
